# Complete chloroplast genome sequence of the medicinal plant ramie (*Boehmeria nivea* L. gaud) and its phylogenetic relationship to other Urticaceae species

**DOI:** 10.1080/23802359.2021.1878959

**Published:** 2021-03-22

**Authors:** Xiangyu He, Zihong Zheng, Qirui Wang, Manjia Zhou, Guanghui Liao, Yuqing Ge, Rubin Cheng

**Affiliations:** a College of Pharmaceutical Science, Zhejiang Chinese Medical University, Hangzhou, China; b The Administration Bureau of Zhejiang, Jiulongshan National Nature Reserve, Suichang; c The First Affiliated Hospital of Zhejiang, Chinese Medical University, Hangzhou, China

**Keywords:** *Boehmeria nivea*, complete chloroplast genome, phylogenetic analysis, Urticaceae family

## Abstract

Ramie (*Boehmeria nivea* L. Gaud) is a traditional fiber crop and important medicinal plant belonging to the family Urticaceae. In this study, we determine the complete chloroplast genome sequence of *B. nivea*. The assembled chloroplast genome is 156065 bp in length and shares the conserved quadripartite structure as other cp genomes in *Boehmeria*. The genome contains 131 genes, including 84 protein genes, 8 rRNA genes, 37 tRNA genes and 2 pseudo genes. There are 17 duplicated genes in the IR region. The overall GC content of *B. nivea* is 36.33%, with the highest GC content of 42.72% in IR region. A total of 67 simple sequence repeats are identified in the cp genome of *B. nivea*. Phylogenetic analysis demonstrated that *B. nivea* clustered together with *B. tomentosa,* further forming a monophyletic group with the species of Debregeasia and Pipturus. This work provides basic genetic resources for developing robust markers and investigating the population genetics diversities for *B. nivea.*

Ramie (*Boehmeria nivea*), also known as China grass, is a perennial herb with high economic values due to its production of natural fiber. It is a widely planted species in south of China. The ramie leaf was also utilized as feed supplement and livestock forage grass since the high content of protein and nutrients (Mu et al. 2020). Furthermore, the leaves and roots of *B. nivea* have been used as traditional Chinese medicine that possesses a variety of pharmacological properties. The ethanol extract of *B. nivea s*uppressed mast cell mediated allergic inflammation, indicating its potential application in the treatment of various allergic disorders (Lim et al. 2020). In addition, the leaf of *B. nivea* exhibited significant anti-hepatitis B virus activity (Wei et al. 2014). It is also reported that *B. nivea* is rich in active polyphenol compounds and exerts potent laxative and antioxidant effects in model rats with loperamide-induced constipation (Lee et al. 2020). The complete chloroplast genome information would contribute to the cultivation research and species identification of this important plant. Here, we report the complete chloroplast genome of *B. nivea* to provide a genomic resource for molecular marker development and clarify the phylogenetic relationship in family Urticaceae.

The sample of *Boehmeria nivea* was collected from Fuyang area of Zhejiang Province (30°05′2.4″N, 119°53′20.4″E). The specimen is deposited at Medicinal Herbarium Center of Zhejiang Chinese Medical University, Hangzhou, China (Voucher Identifying Number ZM-1963). Total genomic DNA was extracted and sequenced using the Illumina Hiseq Platform according to the previous reprot (Gao et al. 2020; Wang et al. 2020). The chloroplast genome of *B. nivea* was assembled by metaSPAdes with the chloroplast sequence of *B. umbrosa* as reference (Nurk et al. 2017). The chloroplast was annotated using GeSeq and further confirmed by BLAST (Tillich et al. 2017). The complete cp genome of *B. nivea* was submitted to GenBank with the accession number of MW057777.

The length of the complete chloroplast genome sequence of *B. nivea* was 156065 bp, with a large single copy (LSC) region of 85901 bp, a small single copy (SSC) region of 18972 bp, and two separated inverted repeated (IR) regions of 25596 bp each. A total of 131 genes were identified in the cp of *B. nivea*, containing 84 protein-coding genes, 37 tRNA genes, 8 rRNA genes and 2 putative pseudo genes. The overall GC content was 36.33%, and the corresponding GC contents for LSC, SSC and IR regions were 34%, 29.61% and 42.72%, respectively. The genome included 17 duplicated genes in the IR region and exhibited 50.23% protein-coding sequences. Moreover, a total of 67 small single repeats (SSR) are identified in the cp of *B. nivea*, ranging from 10 bp to 123 bp.

The complete genome sequences of *B. nivea* and other 10 representative Urticaceae species were analyzed using MEGA 7.0 by maximum-likelihood (ML) method to confirm its phylogenetic position. The result demonstrated a sister relationship between *B. nivea* and *B. tomentosa*, indicating a close genetic relationship between the two species (Figure 1). The monophyletic group of *B. nivea* and *B. tomentosa* clustered with the combined clade of species from genuses Debregeasia and Pipturus, further forming the Group I in the the family Urticaceae (Figure 1). Furthermore, the species of *B. umbrosa* and *B. spicata* formed Group II, but did not display sister relationship with the monophyletic group of *B. nivea* and *B. tomentosa*, indicating further investigation and classification revision in the genus Boehmeria. These results would contribute the development of molecular markers and understanding of evolutionary history and cultivation strategy for *Boehmeria nivea*.

**Figure 1.**
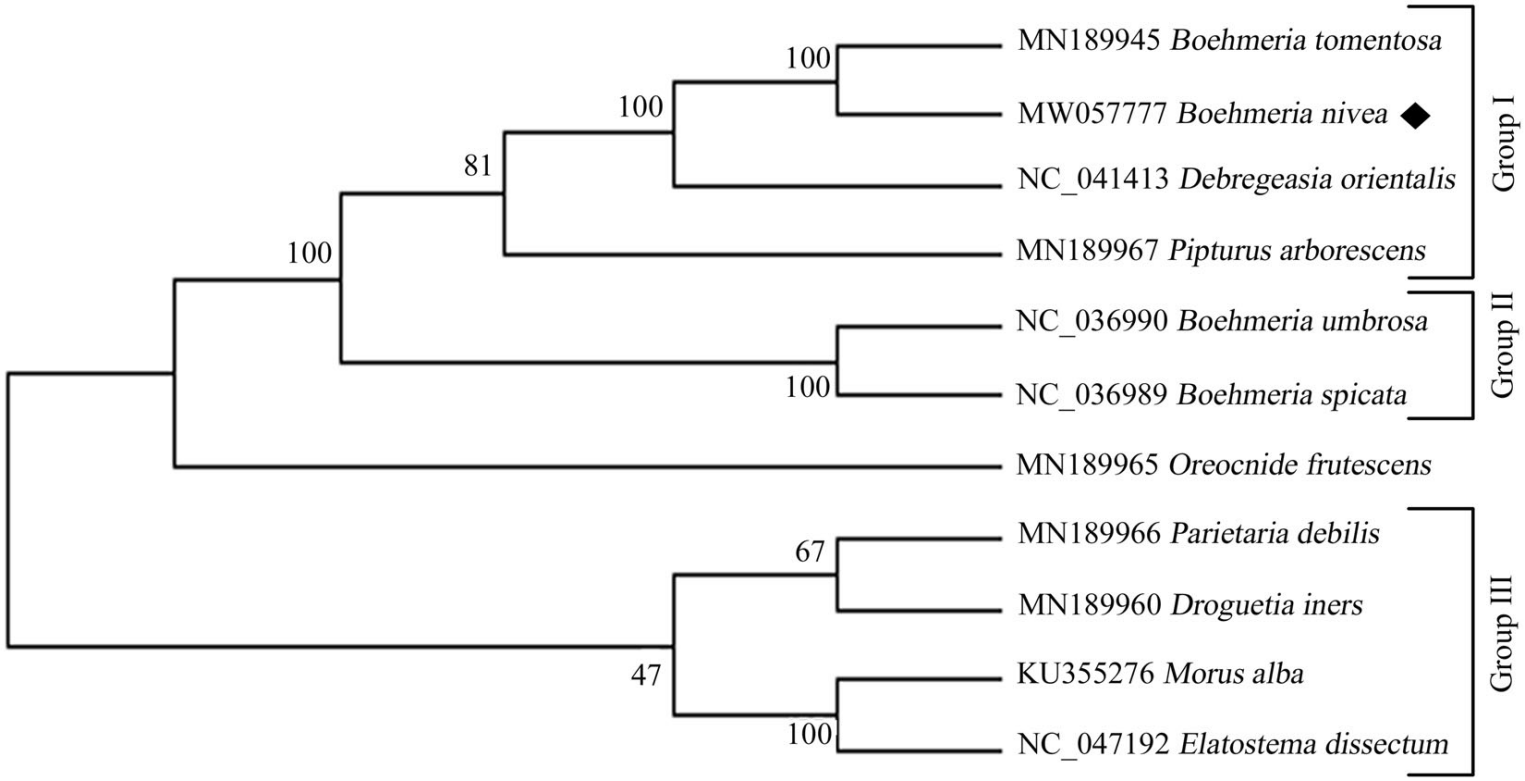
ML phylogenetic tree analysis of *Boehmeria nivea* and other representative Urticaceae species based on the complete chloroplast genome sequences. Numbers on the nodes are bootstrap values from 100 replicates. The GenBank accession numbers were listed before the species name.

## Data Availability

The genome sequence data that support the findings of this study are openly available in GenBank of NCBI at (https://www.ncbi.nlm.nih.gov/) under the accession no. MW057777. The associated BioProject, SRA, and BioSample numbers are PRJNA689705, SRR13357339 and SAMN17215053, respectively.
